# Indirect Inguinal Hernia and Hiatal Hernia in an Elderly Female Cadaver: A Report of a Rare Case

**DOI:** 10.7759/cureus.77327

**Published:** 2025-01-12

**Authors:** Yash Desai, Prutha Patel, McKenzie Lydon, Michael Breiner

**Affiliations:** 1 Anatomy/Surgery, Edward Via College of Osteopathic Medicine, Blacksburg, USA; 2 Surgery, Edward Via College of Osteopathic Medicine, Blacksburg, USA

**Keywords:** cadaver case report, deep inguinal ring, hesselbach's triangle, hiatal hernia, indirect, inguinal hernia, intra-abdominal pressure

## Abstract

This case report highlights the rare co-occurrence of an indirect inguinal hernia and a hiatal hernia identified in a 92-year-old female formalin-fixed whole-body donor. While indirect inguinal hernias are relatively uncommon in women, the simultaneous presence of a hiatal hernia adds to the uniqueness of this case. This discovery offers an opportunity to explore the anatomical, clinical, and embryological factors contributing to hernia formation, as well as the incidence rates and sex-related disparities in their occurrence. The findings are contextualized within the donor's advanced age and suspected chronic respiratory disease, which likely contributed to increased intra-abdominal pressure and weakened connective tissue integrity. The authors obtained appropriate permissions and approvals for the use of data and images, adhering to institutional ethical guidelines and ensuring compliance with donor consent protocols.

## Introduction

Inguinal hernias occur when abdominal contents move through the peritoneal cavity into the inguinal canal. These hernias are categorized based on their relationship to the inferior epigastric vessels. Direct inguinal hernias protrude medially through the inguinal triangle (Hesselbach’s triangle) with boundaries that consist of the linea semilunaris medially, the inferior epigastric vessels superolaterally, and the inguinal ligament inferiorly [1]. Indirect inguinal hernias, which are more common when compared to direct inguinal hernias, traverse the deep inguinal ring lateral to the inferior epigastric vessels [2]. These pathologies are in large part due to weakened segments of the abdominal wall, which allow passage of the peritoneal contents. Indirect hernias are relatively rare in women due to the narrower inguinal canal and are often associated with conditions that increase intra-abdominal pressure, such as obesity, chronic obstructive pulmonary disease (COPD), and connective tissue disorders [3,4].

Inguinal hernias have a bimodal distribution, with higher prevalence in children and adults around 40 years old [5]. Male patients account for the majority of cases (90%), with female patients representing less than 10% [5]. While this pathology is rather common in men, with a 27% lifetime incidence, it is a rare one in women at approximately 3%, and when present, it is commonly found on the right side [5]. Given its rarity in female patients, particularly in elderly patients, indirect inguinal hernias in women are often underreported. In addition to inguinal hernias, hiatal hernias are another significant type of hernia, especially in the elderly population. A hiatal hernia occurs when a portion of the stomach protrudes through the diaphragm into the thoracic cavity. The incidence of hiatal hernias increases with age, affecting approximately 60% of individuals over 50 years old, with prevalence rising in those aged 70 and older [6,7]. Hiatal hernias are more prevalent in female patients, with a female-to-male ratio of about 2:1 [8]. Factors contributing to the increased incidence in older adults include weakened diaphragm muscles and connective tissue disorders [9]. Additionally, it is worth noting that varying interferences with embryologic events can cause abnormalities in the development of these tissues.

## Case presentation

**Figure 1:**
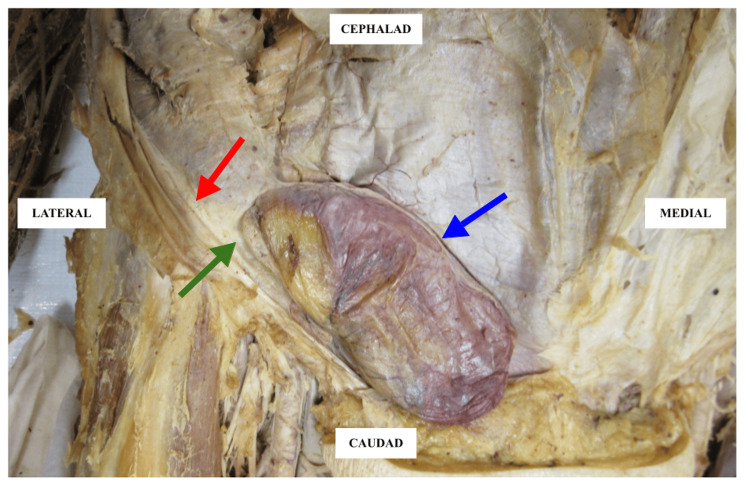
Remarkable finding of abdominal contents protruding through the anterior abdominal wall, highlighting the discovery of an indirect inguinal hernia. Red arrow: inguinal ligament; blue arrow: hernial sac; green arrow: superficial inguinal ring

A 92-year-old female formalin-fixed whole-body donor was selected for dissection as part of the Gross Anatomy Laboratory program at Edward Via College of Osteopathic Medicine in Blacksburg, Virginia. The authors obtained appropriate permissions and approvals for the utilization of data and images, adhering to ethical guidelines and ensuring compliance with donor consent protocols at the institution. The dissection was completed by rising second-year medical students. During the dissection, an abnormal protrusion of abdominal contents was discovered traveling through the anterior abdominal wall on the right side of the donor (Figure 1). Exploration of the left side was also done, which revealed no abnormalities.

**Figure 2:**
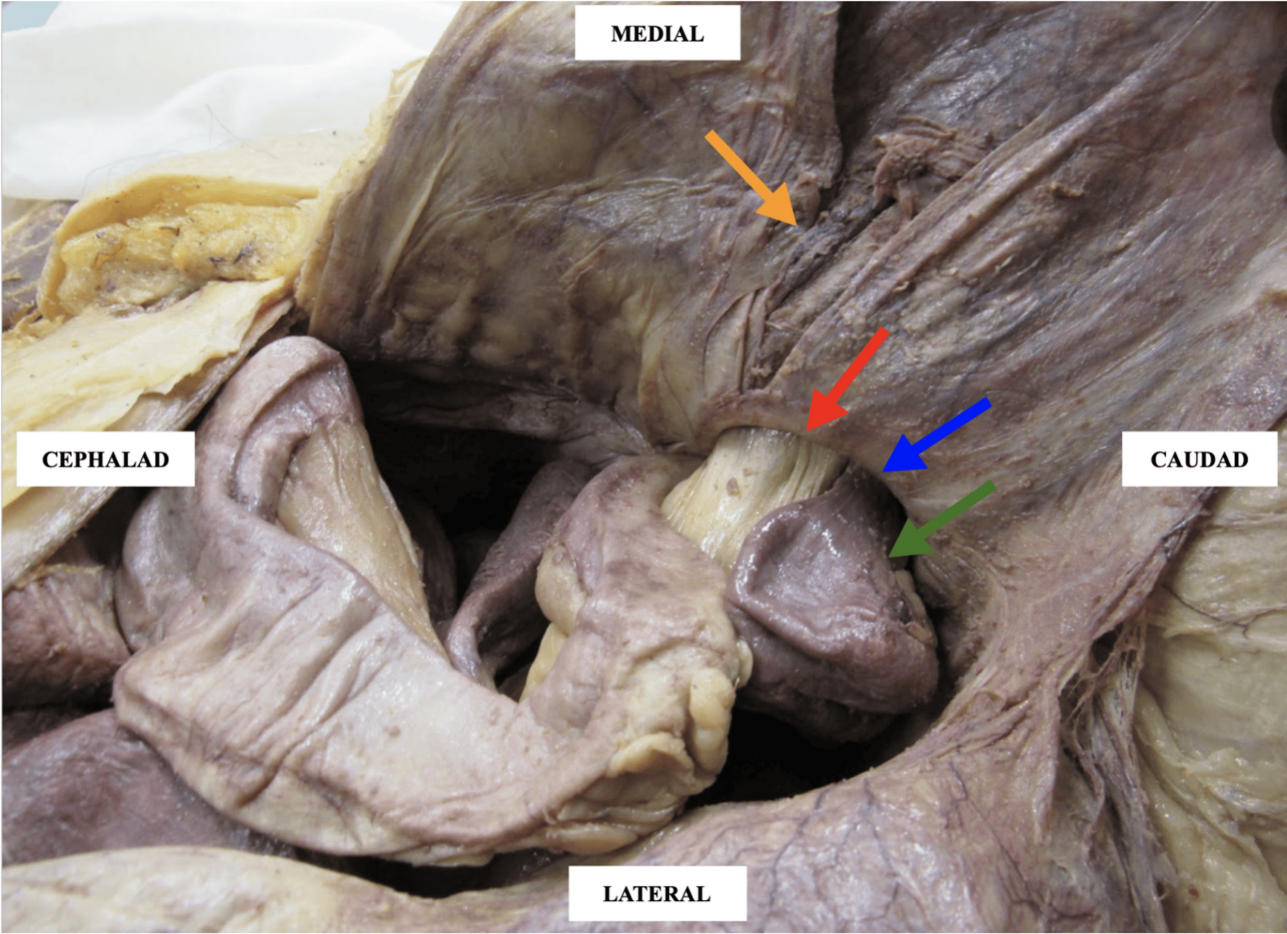
Key anatomical structures involved in the cadaver’s indirect inguinal hernia. Orange arrow: inferior epigastric vessels; red arrow: mesentery; green arrow: proximal ileum; blue arrow: opening of the deep inguinal ring

Upon further inspection, it was discovered that this was an indirect inguinal hernia characterized by the protrusion of peritoneal contents through the internal inguinal ring, lateral to the inferior epigastric vessels (Figure 2). 

**Figure 3:**
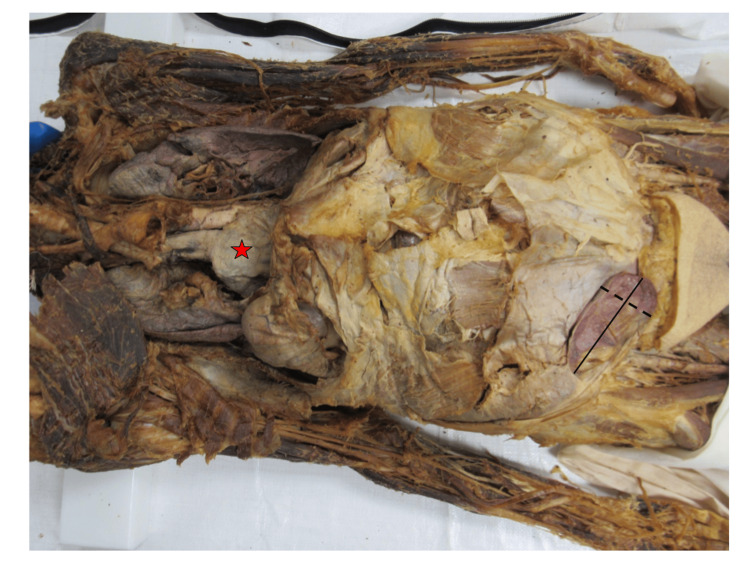
Measurements of the indirect inguinal hernia. The straight line measures 11 cm, from the point of herniation through the abdominal wall to the distal end of the hernia. The dashed line represents the widest part of the hernia, which measured at approximately 4.3 cm. Additionally, the red star indicates the type 3 hiatal hernia.

The hernia was measured to be 11 cm from the point of herniation through the abdominal wall to the distal end of the hernia. It measured approximately 4.3 cm at the widest point of the hernia, which was approximately 1.3 cm from the distal aspect of the hernia (Figure 3).

The hernial sac contained mesentery and a segment of the proximal ileum. The hernial sac showed slight congestion, yet there were no indications of strangulation or blockage. During the dissection of the foregut of the donor, a type 3 hiatal hernia was also discovered on the left side of the donor (Figure 3). The hernia was composed of parts of the stomach, largely the fundus of the stomach, and herniated through the abdominal diaphragm, lateral to the esophagus. Hiatal hernias, as seen in Figure 3, and inguinal hernias co-occur more frequently than would be anticipated by random chance. The general risk of an inguinal hernia in individuals with a hiatal hernia is 2.5 times higher compared to those without one [10]. This relationship is seen due to similar underlying factors, such as increased intra-abdominal pressure and connective tissue disorders [10,11].

## Discussion

In the overall population, inguinal hernias are the most common types of hernias and are accurately denoted as "groin hernias" due to their location. The term "groin hernia" has a longstanding history of describing hernias occurring in the inguinal region, specifically within the groin. Henri Fruchaud, a French surgeon of the 20th century, played a pivotal role in advancing the categorization and surgical management of hernias. His efforts, especially within the field of hernia surgery, significantly enhanced the understanding of these conditions [12]. Franz K. Hesselbach, a German surgeon and anatomist of the 19th century, coined “Hesselbach’s triangle” denoting the area where a specific type of hernia occurs [12]. This hernia was later described as a direct inguinal hernia, leading to the categorization of the hernia that occurs lateral to the inferior epigastric vessels as an indirect inguinal hernia.

As discussed previously, inguinal hernias are more prevalent in men than women, with the more common type being indirect inguinal hernias accounting for approximately two-thirds of all inguinal hernias [13]. Although direct inguinal hernias in both sexes are primarily caused by age-related stress and weakened abdominal muscles of the inguinal canal, the difference in the incidence of indirect inguinal hernias based on biological sex is due in large part to the descent of the testes during development [1]. Defects in this embryological process lead to an increased incidence of indirect inguinal hernias in male patients [1]. In contrast, intersex differentiation syndromes are associated with an increased incidence of inguinal hernias in genetically female individuals due to abnormal genital development [14].

Due to their close anatomical proximity, the two subtypes of inguinal hernias may present similarly on physical examination. The symptoms and history of present illness generally overlap, making it rather difficult to differentiate the two subtypes clinically. The patient’s history of present illness generally consists of sudden onset of severe pain with or without sepsis; pain that may worsen with Valsalva maneuver, coughing, or other physical stressors; symptoms that are worse at the end of the day; and relief of symptoms when lying down or manually reducing the hernia. However, about one-third of patients presenting with inguinal hernias report being asymptomatic [15].

The primary complications of inguinal hernias are the progression of a reducible hernia to an incarcerated or strangulated hernia [16]. The likelihood of strangulation occurring is 0.29% for all cases of inguinal hernias [17]. Among strangulated hernias, indirect inguinal hernias are found to be predominant due to their narrow neck as compared to direct inguinal hernias [18]. Due to this commonly encountered progression, the critical need for timely detection is highlighted.

Treatment options for inguinal hernias encompass a range of choices, including watchful waiting, open repair, and laparoscopic repair. The selection of the appropriate intervention should be tailored to the patient's age, underlying health conditions, and anatomical factors. Surgery is often the preferred route for treatment and is generally only avoided in asymptomatic and non-progressive inguinal hernias in male patients [19]. Open approaches have long been the staple of inguinal repairs and include tissue repairs and prosthetic repairs. In contrast to open repairs, laparoscopic repairs have been shown to decrease recurrence rates and improve postoperative pain, leading to patients being able to resume normal activities sooner [19]. The primary laparoscopic repairs used include the transabdominal preperitoneal (TAPP) procedure and the total extraperitoneal (TEP) procedure. The primary complication in hernia repairs, both laparoscopic and open, is that there is potential for causing damage to the intraperitoneal viscera and structures [19].

Hiatal hernias occur when parts of the gastrointestinal tract herniate through the diaphragmatic esophageal hiatus into the thoracic cavity [9]. There are four types of hiatal hernias, and as mentioned previously, this donor had a type 3 hiatal hernia. These types are classified based on the degree of herniation and the structures involved [9]. Type 1 (sliding hiatal hernia) is the most common type and involves the gastroesophageal junction sliding superiorly toward the thoracic cavity [9]. Type 2 (paraesophageal hernia) involves the stomach herniating alongside the esophagus with the gastroesophageal junction remaining in place [9]. Type 3 is a combination of the previous two where both the gastroesophageal junction and a portion of the stomach herniate into the chest [9]. Type 4 hiatal hernias involve other abdominal organs in addition to the stomach [9]. Although hiatal hernias have an incidence of about 55%-60% in the population above 50, the vast majority are type 1 with the other three types only comprising 5%-15% together [9,20].

The unrepaired indirect inguinal hernia in the 92-year-old female donor is a very unusual finding as these hernias are generally repaired, especially considering the size of the hernia in question. According to the pathology report, the cause of death in the donor was acute hypoxic respiratory failure (AHRF) and lactic acidosis. Upon examination of the donor’s lungs, signs of COPD were found, which explains the findings on the pathology report. These lung pathologies are suspected to be the primary cause of the two hernias seen in this donor due to the increased intra-abdominal pressure. COPD is linked to frequent coughing, which exerts considerable stress on the abdominal wall over time, weakening the musculature and facilitating herniation [3]. This chronic respiratory condition not only exacerbates existing hernias but can also contribute to their initial formation, especially in individuals with already compromised tissue integrity [3]. Connective tissue disorders are also known to increase susceptibility to hernias [4]. Genetic conditions characterized by defects in collagen synthesis cause weakened structural support of the abdominal wall, predisposing affected individuals to both inguinal and hiatal hernias [4]. This donor's age and possible underlying connective tissue pathology may have contributed to the development of both hernias, warranting further consideration when evaluating patients with multiple hernias, particularly in those without common risk factors.

## Conclusions

Despite the limited access to the donor’s medical history, this case provides valuable insights into the potential mechanisms underlying the development of both an indirect inguinal hernia and a hiatal hernia in an elderly female donor. The concurrent presence of these hernias highlights the importance of considering rare anatomical findings in women and underscores the role of conditions such as chronic respiratory diseases and connective tissue disorders in predisposing individuals to hernia formation. This case serves as a reminder of the need for a comprehensive understanding of hernia etiology and the interplay of anatomical, clinical, and pathological factors. Further exploration of these relationships could inform both diagnostic approaches and management strategies for hernias, particularly in populations with atypical presentations.
